# Developing Speaking Skills in Third-Grade Students Through the Analysis of Visual Material in Two Languages (Lithuanian and English)

**DOI:** 10.3390/bs15101362

**Published:** 2025-10-05

**Authors:** Daiva Jakavonytė-Staškuvienė, Guostė Streikutė

**Affiliations:** Education Academy, Vytautas Magnus University, 03113 Vilniaus, Lithuania

**Keywords:** primary education, bilingual education, speaking skills, visual material

## Abstract

In language classes, speaking skills are often taken for granted, and not enough attention is paid to developing these skills in a targeted way. In our study, the speaking skills of third-grade students (N = 46) are developed in integrated Lithuanian and English lessons through the analysis of visual material. Visual material is an aid and a means for expanding students’ vocabulary and developing their ability to express their thoughts verbally. The students are aged 9–10 years old. The aim of the study was to investigate the development of third-grade students’ speaking skills using visual material analysis in two languages. The Action Research was conducted in a school in one of Lithuania’s major cities. During the Action Research, students completed mind maps and analyzed visual material by answering questions in two languages. The questions were designed to cover different groups of thinking skills (knowledge and understanding, drawing conclusions, interpretation, and evaluation). The students spoke their prepared answers to the questions. The accuracy and correctness of the answers, English pronunciation, and the ability to speak in complete sentences were evaluated.

## 1. Introduction

Language skills are an integral part of smooth communication, helping to ensure a high quality of life and career opportunities. In the globalized world we live in, people increasingly speak more than one language (Suarez & Beato, 2023). Bilingualism and its importance are becoming increasingly relevant, especially since English surrounds students not only when traveling, but also in their immediate environment when searching for information on the Internet, choosing goods in a store, or playing games, reading, and watching movies in their free time. Knowledge and learning of English have become an important skill (Kiruthiga & Christopher, 2022). In addition, the European Commission (2018) has identified multilingualism as a key competence for all European citizens (*Key Competences for Lifelong Learning in the European Schools*, 2018). Multilingualism is understood as the ability to speak more than two languages, which must be developed from the early school years.

The aim is to find teaching methods that will help students master languages through practical activities. One such method is learning from the immediate environment, turning it into a language learning tool. This is also emphasized in Lithuania’s general programs, which define mediation as the communication of the content of various texts (both visual and written) to other people, helping them to understand the thoughts of others (*Lithuanian General Program for the First Foreign Language*, 2022). Although, in theory, models of simultaneous bilingual education are emphasized in documents defining educational content, there is a lack of research in this area. In particular, there is a lack of research that would substantiate issues of bilingual education didactics at an early age.

Mediation training encompasses not only English language skills (writing, reading, listening, and speaking), but also all the same Lithuanian language skills, as students use both languages together to achieve a communicative goal—to help someone who does not know the language understand the context of a written or visual text. Various studies show that primary school students benefit greatly from analyzing audiovisual texts (i.e., watching films or film excerpts) (Avello & Muñoz, 2023). Films are the most effective audiovisual tools for understanding language culture, as they allow us not only to see and hear the language, but also to discover its more complex structures, such as various idioms and wordplay. (Aksu Ataç & Günay-Köprülü, 2018). Visual text analysis is useful for developing both native and foreign languages. Multimodal education allows children to discuss, speak, and listen, combining words, thoughts, and images in class (Petrošienė, 2022). This means that when watching a film, students engage many senses, and when analyzing it, they develop more than one language skill. The development of Lithuanian language skills through films can even be understood as “information reception through multiple senses” (Cicėnaitė-Milaševičiūtė & Juškevičienė, 2020). English language skills in the analysis of visual material also show great promise. When it comes to language learning, films have the potential to bring language off the page and into the classroom, contextualizing its use in an interesting and accessible way (Bottomley, 2018). The opportunity to analyze and talk about a film develops students’ speaking skills through activities that interest them, allows them to identify with the characters, and hear real language, i.e., voices and pronunciation (Roslim et al., 2021). In our study, the analysis of visual material was conducted in two languages: the students watched the film in one language and prepared their answers to the questions in two languages (half of the questions were answered in Lithuanian and the other half in English).

Research question: how does the analysis of visual material in two languages develop the speaking skills of third-grade students?

The aim of the study: to investigate the development of third-grade students’ speaking skills using visual material analysis in two languages.

In order to achieve this aim, the following study objectives were set:

1. To examine the scientific literature on the analysis of visual material in terms of content analysis in two languages in primary school.
2. To reveal how to develop students’ skills in two languages (Lithuanian as their native language and English as a foreign language) during a single lesson, when the material is presented in one language but analyzed in two languages.
3. Describe students’ experiences in developing bilingual skills based on an analysis of visual material.

## 2. Theoretical Basis for Developing the Speaking Skills of Third-Grade Students Using Visual Material Analysis in Two Languages

### 2.1. Developing English as a Second Language Skills in Third Grade

The document *Lithuanian General Program for the First Foreign Language* (2022) highlights achievements such as understanding and creating audiovisual texts, language interaction in virtual space, and mediation. Students developing their English language skills are empowered to become mediators—able to use English to retell a video they have seen or a text they have analyzed in Lithuanian, and vice versa—to convey material they have watched or read in English in Lithuanian. This skill is not only relevant in today’s world, but also prepares students for their careers. International relations are becoming increasingly important, and good translation from one language to another can be applied in various fields of work (S. Ahmad, 2015).

Vocabulary is one of the most important components of foreign language comprehension and mastery (Suleiman Al Qunayeer, 2021). In addition, a broad vocabulary is a strong advantage in English reading (Oh et al., 2023), while a lack of vocabulary can be an obstacle to the effective development of listening skills (Salape et al., 2023). Speaking is not just about pronouncing words; it involves conveying one’s thoughts through words (Kiruthiga & Christopher, 2022). It is also clear that vocabulary plays an important role in students’ writing (Baba, 2009; Lee & Muncie, 2006; Olinghouse & Wilson, 2013; Wang, 2023). All language skills are developed through the verbal analysis of subject matter is explaining concepts and their meanings, analyzing the grammatical structure of words (Jakavonytė-Staškuvienė & Žemgulienė, 2016). From this, it can be concluded that vocabulary is included and important in developing all the skills defined in the programs. In the early grades, students learn to use frequently heard everyday words, express concepts, and describe thoughts or feelings in phrases and sentences. In other words, students must master certain concepts that will help them communicate fluently in a foreign language in everyday situations. Table 1 presents the abstract concepts that students must master in grades 3–4.

After analyzing the abstract concepts presented, it can be said that students in grades 3–4 need to have a relatively large vocabulary and be able to use concepts in various contexts. As already mentioned, vocabulary learning is an essential aspect of language teaching and learning (Yangin Ersanli, 2023). Only by mastering vocabulary can one improve in other areas. However, the ability to learn vocabulary is one thing, and the skill of applying it at the right time and in the right situation involves other, deeper abilities.

One of the areas of achievement that will be explored in more detail in this paper is the production of spoken text, or in other words, speaking. Regardless of whether the language is native or foreign, speaking is the primary means of communication that we use every day. As a foreign language skill, speaking is widely considered one of the four most important language skills because it is the primary means of communicating and establishing relationships with others (Vu, 2023). Communication is the most important English language skill (according to the document *Lithuanian General Program for the First Foreign Language*, 2022). Speaking reveals a student’s ability to use their vocabulary, general understanding of the language, ability to communicate, and achieve communicative goals. Speaking is both an active and productive skill (Kiruthiga & Christopher, 2022) because it involves the use of vocabulary and the combination of language sounds.

Another important skill emphasized in the general programs is text (written, visual) mediation. The term *mediation* is defined in the program as the oral or written transmission of specific information (visual material, messages, statements, text summaries) (*Lithuanian General Program for the First Foreign Language*, 2022). Mediation is explained as the transfer of text content to another person who does not have access to it due to linguistic, cultural, semantic, or technical barriers (*Key Competences for Lifelong Learning in the European Schools*, 2018). Third-grade students learn according to level A1, and mediation at this level includes two essential aspects: the use of simple words or signs and nonverbal signals to show that the learner is interested and understands the idea, and the presentation of the analyzed text in short, simple signs, sentences, and notes, posters.

It is important to note that developing speaking or mediation skills is a considerable challenge for teachers, as students often encounter various negative attitudes towards speaking. Such factors may include a bad attitude, negative beliefs about speaking a foreign language, and difficulties in using the vocabulary of the second language (Güneş & Sarıgöz, 2021). In addition, reluctance to express thoughts verbally is influenced by self-doubt, anxiety, fear of failure, shyness, and lack of motivation (Kiruthiga & Christopher, 2022). The broader the vocabulary, the more coherently and clearly a language user can express their thoughts and communicate more fluently (Antar, 2023). The solution to a situation where a student does not want to express themselves verbally is to try and apply appropriate methods, create a supportive learning environment, and provide opportunities for integration that help develop English language skills in other subjects. It is important to include more speaking practice in the educational process because the more practice students have, the more confident they become in their abilities.

### 2.2. Developing the Lithuanian Language Skills of 3rd Grade Students as Their First Language

The primary, dominant language (in this case, Lithuanian) that a child knows is the everyday means of communication, passed down from generation to generation, surrounding every Lithuanian from an early age. It is needed not only in Lithuanian language lessons, but also in other subjects: when reading texts or instructions, and when performing various tasks. It can even be said that without a proper command of the Lithuanian language, it is difficult to improve in other subjects. Language can be described as an independent learning tool, as evidenced by such student skills as the ability to analyze, reason, and summarize information, as well as openness to learning (Mereckaitė, 2022). This means that by knowing Lithuanian well, students can feel more confident in other subjects is they use language as a tool to help them better understand the conditions, learn the content, and express their knowledge. Since this paper will focus mainly on speaking skills, it is important to emphasize that communication competence dominates the document *Lithuanian Language and Literature General Program for Primary Education* (2022) (as it does in English). Speaking skills cover three main areas: speaking according to purpose, situation, and audience; use of linguistic means of expression; and application of speaking strategies. Each of these areas defines what a student who has reached a higher level of achievement should apply when speaking. In the A3 achievement area, the aspects of language correctness, originality, and richness are emphasized, while in A4, reflection on one’s own speech is emphasized. Achievement area A2 reflects aspects of linguistic culture, accuracy, and expressiveness. This is perhaps the most broadly described area, revealing three factors that learners must take into account when developing their speaking skills: the purpose of speaking (the learner considers why they need to speak, for example, to ask for something, to obtain or convey information, to communicate, etc.); the speaking situation (the learner pays attention to whether the situation is formal or informal); the addressee (the learner responds to who they are talking to: a peer or an adult, a close person or a stranger) (see Table 2).

Analyzing the content of Table 2, we see that the aim is for students to value speaking as one of the most important steps in the communication process. According to the general program, students must develop both dialog and monolog speaking skills. Dialog includes conversations on relevant topics in the classroom or in a virtual space, in pairs or in groups. Emphasis is placed on the importance of formulating logical questions that are relevant to the topic of conversation and providing coherent answers. When speaking in monologs, students must be able to interest, inform, describe, etc. This means that by the third grade, students should have mastered all aspects of speaking: taking context into account, using correct language, engaging in dialog in a purposeful manner, expressing their thoughts in monologs, and reflecting on the success or failure of their speech. Speaking is the most effective means of communicating one’s ideas, opinions, and views to others (Kiruthiga & Christopher, 2022). The importance of this skill was also emphasized by the founder of sociocultural theory, Vygotsky (1978), who argued that social interaction is the essence of cognitive development (*Vygotsky’s Theory—An Overview Science Direct Topics*, 2020). He emphasized the interdependence of language and thought, and his theoretical concepts laid the foundation for constructivism. Since this theory emphasizes the importance of speech as a skill, it is worth analyzing different sources that have examined sociocultural development theory. According to Vygotsky’s theory, language is considered an essential tool for communication, and human culture is understood precisely through speech. Social interaction helps children develop their ability to use language (*Vygotsky’s Theory—An Overview Science Direct Topics*, 2020). From this, we can conclude that in this theory, language is even perceived as a reflection of an individual’s culture, which must be developed through constant communication. This means that the more social interaction a child has, the more their speaking ability is developed. Vygotsky’s sociocultural theory also proves that language is a tool for the growth of social intelligence, and that speaking and its development from an early age provide an opportunity to expand students’ cultural awareness and allow them to express themselves in all areas of communication.

### 2.3. Bilingualism and Its Role in Developing Students’ Language Skills

Bilingualism is the ability to speak and use two or more languages in everyday communication (*Universal Lithuanian Encyclopedia*, 2025). Students who have mastered more than one language are better able to focus on important information and ignore irrelevant and distracting stimuli, as they are accustomed to following two different sets of rules. For this reason, bilingual students learn to become critical thinkers and perform better at problem-solving tasks (Elt, 2015; Vilkienė, 2011). The integration of languages and other subjects develops higher thinking skills in students and turns the learning process into active linguistic activity (Jakavonytė-Staškuvienė & Žemgulienė, 2016). Such language teaching helps students to better master not only their native language but also foreign languages. First language intervention plays an important role in second language learning (Biglari & Struys, 2021). Without knowledge of the native language and its correct use, the development of a second language would be difficult to achieve. When two languages are used in the same lesson, this provides more opportunities for language learning and meaning formation (Davis, 2023; Ergül, 2023).

In bilingual education, it is very important to take into account the age of the students. In order to better understand the language development of children of this age, it is worth considering the child’s developmental abilities (Rakickienė, 2021). As mentioned above, third-grade students are usually 8–9 years old. According to this description, 7–8-year-old children are able to rely on their inner language, use more varied and complex grammatical structures, understand more concepts, use synonyms, classify them, and their vocabulary is expanding rapidly. The language of 9–10-year-olds is even more advanced. Vocabulary expansion becomes less and less related to the child’s direct experience; a definition is enough for them to understand and incorporate a new word into their language. Ambiguities (metaphors, figurative meanings) are even easier to understand, and children are able to use them in humor. Thus, in both age groups, rapid vocabulary expansion and better understanding of concepts and other linguistic structures are emphasized, so it can be concluded that students already have a good understanding of concepts and simple metaphors in their native language and are able to use them in everyday life. Knowing what a child should be able to do according to their age allows for more effective foreign language teaching, as it is then clear what the child already uses and understands in their own language according to their age.

### 2.4. Methodology for Analyzing Visual Materials in Developing Students’ Language Skills

Living in the digital age, we perceive various visual stimuli as part of everyday life. Watching films, videos, and other visual material is a common way for students to spend their free time, which brings them joy and arouses their interest. Watching films encourages emotional engagement, connects students with characters, encourages them to understand their thoughts, feelings, and motivations, and teaches them to overcome preconceived biases and view situations critically (Djamàa, 2018). Given the students’ engagement, it is worth exploring how this enjoyable activity could be applied in the educational process. Films contain wise scenes and interesting images that are suitable for language learning, and it is also expected that students can enrich their vocabulary through films (Hestiana & Anita, 2022). From visual material, students can hear how language is actually used in real situations and gain valuable insight into the culture in which the native or foreign language is spoken (Ashcroft et al., 2018). By seeing images and hearing the characters talk, students not only obtain a better sense of what the language means, but also remember words more easily, word usage in a specific context (Ashcroft et al., 2018).

The analysis of photographs, illustrations, and videos should encourage open discussions and verbal commentary on the content (Mat Halif et al., 2020). Visually, students can see the various reactions, moods, facial expressions, and emotions of the characters, and by listening, they can hear the phrases spoken by the characters, their pronunciation, and intonation. Therefore, audiovisual material helps learners develop their speaking skills (Kathirvel & Hashim, 2020). If students are asked to retell the plot of a film or comment on the characters, they develop not only their speaking skills but also more complex thinking skills, i.e., analysis and interpretation. Visual expression tools are taken into account in the Lithuanian general programs (2022) (see Table 3).

The analysis of visual material helps to develop vocabulary. Films are rich in context, which is important for discussing and analyzing the meanings of words, and the animations used, combined with interesting content, draw learners’ attention to the use of certain words in different contexts and encourage word recall (Rashtchi et al., 2021). When watching a film, regardless of the language, students hear many new words, their use in different situations, and hear them pronounced correctly. Therefore, video analysis can be treated as a learning support (Li, 2025). This helps them to better memorize vocabulary, which is a fundamental language skill. The use of audiovisual media is practical and useful for developing speaking skills, as such media convey material to students through hearing and sight, which is more useful than analyzing text through only one of the senses, and also encourages students to communicate with each other verbally and express their ideas (Ampa et al., 2013; Yani, 2022). The analysis of visual material not only teaches students the language through different experiences, but also encourages them to actively discuss, delve deeper, and thus develop their speaking skills. Films are an authentic source from which students can hear real language and see its real-life use, from everyday phrases to eloquent, authentic statements (*Using Film and Media in the Language Classroom: Reflections on Research-led Teaching*, 2019). By analyzing films, students become true language explorers, mastering language through experience—listening, applying and presenting it in different real-life situations. Activities carried out before watching the film help students prepare to understand the story and the characters involved. The tasks given after the film are very important because they allow the student and teacher to assess whether watching the film was worthwhile, and they also improve writing and speaking skills (Alluri, 2018). In addition, visual material develops students’ basic cognitive abilities and lays the foundations for further learning (Lo & Wang, 2024).

We would like to emphasize the importance of using mind maps, as this method helps children to systematize their knowledge and organize familiar words into structures (Fu & Relyea, 2024; Swinburne Romine et al., 2025; Qi & Jiang, 2021). Graphic representation is most suitable for children with average language skills (Qi & Jiang, 2021). This means that it is an appropriate tool for developing English language skills as a foreign language in primary school. Mind maps help to involve students in the learning process, as they actively think and justify concepts based on cognitive content when creating maps (Swinburne Romine et al., 2025). The use of mind maps helps students overcome learning difficulties (Fu & Relyea, 2024).

Our study aims to analyze the pedagogical efficacy of having learners analyze film content in two different languages after having watched a film in just one language.

The following questions are raised:

- How do primary school students react to activities where English language skills are combined with Lithuanian language skills (when part of the lesson is conducted in one language and the other part in another language)?
- What tasks do children manage to perform most effectively, and in what cases is it difficult to express an idea in a particular language?

## 3. Methodology

### 3.1. Characteristics of the Study Participants

A total of 46 students in the third grade participated in the study, including 23 boys and 23 girls. The children were 9–10 years old and had been learning English for two years. Taking into account the lack of development of the students’ speaking skills in both Lithuanian and English, the study was conducted with a focus on developing the students’ speaking skills. The students’ knowledge of English in a general context is strong, most of them have a broad vocabulary, quickly understand new concepts and grammatical structures, and read fluently. The students’ knowledge of Lithuanian is also advanced. However, they rarely express their ideas verbally in either English or Lithuanian. During lessons, students mostly read and write. More than half of the students who participated in the study have basic language skills, one-third have achieved a higher level of proficiency, and 20% are at a satisfactory or threshold level, where children perform activities with the help of a teacher. The English language level of the students who participated in the study is A1, but some of the children have independently mastered the vocabulary of level A2. Prior to this study, the students had no experience of analyzing films orally in English and Lithuanian. This study was their first such experience. There were no students with special educational needs in this study, and the native language of all students is Lithuanian. The school is located in one of the city’s residential areas, where most of the middle class population lives. The school environment is safe, and only 5 percent of the children in the entire school come from socially disadvantaged families. The children have good learning conditions both at school and at home. All children participate in various after-school activities.

### 3.2. Research Methodology and Organization

**Figure 1:**
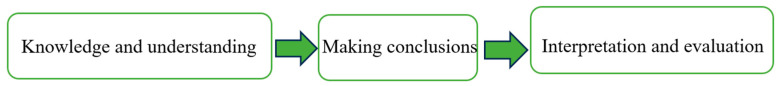
Logical sequence of question formulation (according to Bloom et al., 1956; Anderson & Krathwohl, 2001).

The tasks (instruments) of the organized study are based on Bloom’s taxonomy of levels of task complexity (Bloom et al., 1956; Anderson & Krathwohl, 2001). One of the main ideas of constructivism is that students learn best when they are actively involved in activities (Kurt, 2021; Woolfolk, 1993). This theory argues that students can develop the ability to express their thoughts fluently by actively participating in class: by participating, answering questions, and discussing. The role of students is to master the subject matter through experience and reflection (Aljohani, 2017; Suhendi & Purwarno, 2018). Bloom’s taxonomy provides an appropriate morphological method for creating questions based on different stages of learning. The complexity of the instrument developed for the study questions that students answered verbally created according to the scheme presented in Figure 1. By using well-thought-out questions, teachers can not only obtain factual information, but also help learners connect concepts, draw conclusions, increase awareness, and encourage creative and constructive thinking (Jayakodi et al., 2016). These skills are essential if the teacher’s goal is to improve students’ speaking skills. Due to these aspects, the questions for the study were based on Bloom’s taxonomy (Bloom et al., 1956; Anderson & Krathwohl, 2001), as the main objective of the study was to investigate students’ speaking skills (see Figure 1).

Each level has broader characteristics, which were used as a basis for developing the research instrument. Knowledge and understanding are the simplest questions, the answers to which students can find directly and accurately in the video. In our study, knowledge and understanding encompassed the use of precise words and their correct pronunciation in English. Drawing conclusions is a type of question where part of the answer is in the visual material being viewed, but students must formulate and discover the exact answer themselves. In addition, conclusions also include the generation of ideas and their expression in synonymous words. Interpretation and evaluation are questions that require higher-order thinking skills, personal experience, application of knowledge, and critical evaluation. Interpretation and evaluation include students’ verbal responses when children share their experiences and insights, are able to argue their points, and their thoughts are appropriate to the specific context.

**Figure 2:**
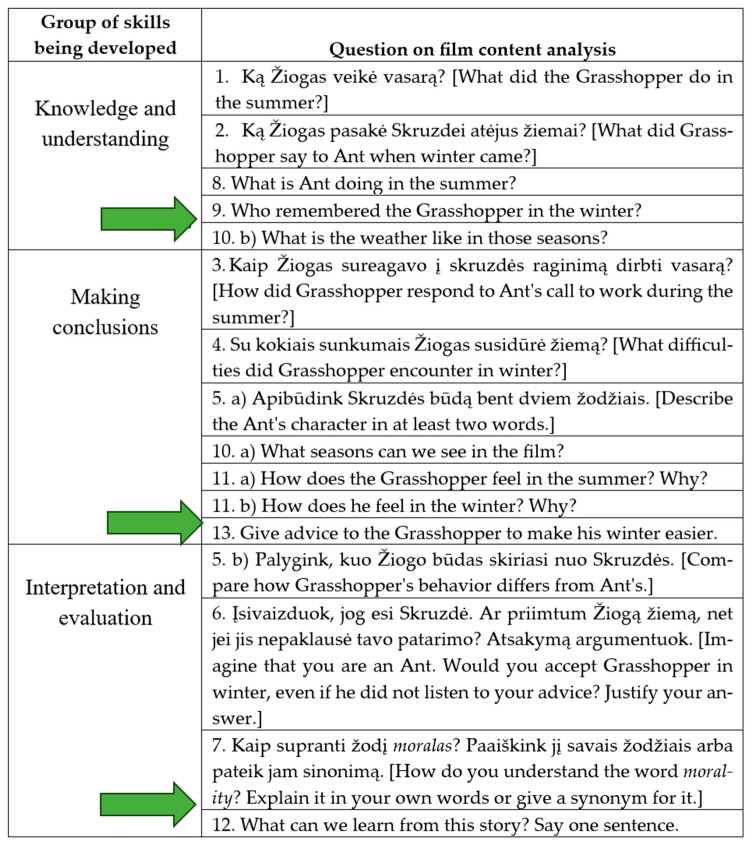
1 Integrated bilingual (Lithuanian and English) lesson questions/tasks divided by skills groups (according to Bloom et al., 1956).

During all lessons of the study, filmed material was analyzed. These were tasks related to the content of the film, of which there were between 10 and 13. Questions were presented to the children according to the sequence of the plot. Each question corresponded to a specific level of Bloom et al. (1956) taxonomy (see Figure 2).

Figure 2 shows that the film analysis questions were distributed across all skill groups. Interpretation and evaluation questions were not presented in English because the students’ vocabulary had not yet reached the level of expression required in a foreign language.

Research method: action research (Carignan et al., 2016; Eden & Ackermann, 2018). This research method was chosen because of the desire to work with students, observe their reactions in real time, and assess the application of knowledge acquired during educational activities and the development of speaking skills. The pedagogical activity was researched in the following way: third-grade students participated in integrated lessons in English and Lithuanian, analyzed visual material (short films, fairy tales, stories), and responded verbally to audiovisual material. English, and integrated lessons of these languages, analyzed visual material (short films, fairy tales, stories), and verbally answered questions about audiovisual perception, which were compiled according to Bloom’s taxonomy, and after each activity, they filled out reflections. The researchers observed the difficulties and successes experienced by the students and took them into account, supplementing the study with new methods, more diverse reflections, and a stronger support system (for English).

Research data was collected through formal observation (Richards & Farrell, 2005; Z. Ahmad, 2020). This study observed how students react, perceive, and evaluate the tasks presented so that they can be adjusted and adapted during the study to ensure that students gain as much benefit and knowledge as possible from the activities. The author of the study recorded the children’s reactions to the activities in her notes. Six lessons were conducted, the sequence and methods used were as follows: Lithuanian language lesson (individual work), English language lesson (individual work), integrated English and Lithuanian language lesson (pair work), integrated English and Lithuanian language lesson (pair work), Lithuanian language lesson (individual work), English language lesson (individual work).

Qualitative content analysis was chosen for the analysis of the research data (Žydžiūnaitė & Sabaliauskas, 2017). In such a study, the researcher analyzes people’s behavior in a natural environment, interacting directly with the research participants, trying to understand their world, and recording their opinions. In this case, the natural environment was the classroom during lesson time, and the researcher carefully observed and evaluated the behavior of the research participants and recorded their responses using a voice recorder.

The study was conducted in a primary school in Vilnius in February and March 2024.

### 3.3. Empirical Research by Stages

#### 3.3.1. Stage 1—Planning the Activity Study

Planning of activities based on an analysis of scientific literature on the development of students’ bilingual speaking skills when analyzing visual material. Six lessons were organized in which students developed their speaking skills:

✓ Lithuanian language lesson (individual work).
✓ English language lesson (individual work).
✓ Lithuanian language lesson (individual work).
✓ English lesson (individual work).
✓ Integrated English and Lithuanian lesson (pair work).
✓ Integrated English and Lithuanian lesson (pair work).

First of all, after reflecting on the content of Lithuanian and English lessons, it was established that too little time is allocated to the development of speaking skills during these lessons. Criteria were then established for selecting visual and audio material that students would listen to and then speak about based on questions related to the content. The material for analysis was selected according to the following criteria: *clear language, short recording length (up to 3 min), good image quality, and a main idea that encourages discussion*. Six short films were selected in this way, and tasks were created for a series of future lessons.

#### 3.3.2. Stage 2—Educational Process and Data Collection

Implementation of planned activities aimed at developing speaking skills (conducting a cycle of six lessons). Each lesson is observed, data is recorded in reflection diaries, and the content of the activities is reflected upon together with the students. After each activity, the content of the activity was reviewed and adjusted based on the data collected during the observation and the reflections expressed by the students.

The students watched the film selected by the researcher at least twice (if necessary, the film was shown a third time); links to the films are provided in the Appendix A. The research tasks were structured as follows:

1. Filling in mind maps with questions related to the theme of the film watched in class. The maps were used to get the students interested in the topic being analyzed and to encourage them to share their own experiences or views. The students first wrote down their thoughts, but then presented their ideas orally. Mind maps for all six lessons are provided in Appendix A. The questions on the mind map were formulated according to the following principle: if the lesson was in Lithuanian, the question could be more complex, encouraging students to express themselves more broadly. If the lesson was in English, the question was simpler and could be answered in a few words in English, not necessarily in full sentences (taking into account that the students were only in their second year of learning English). When the lesson was integrated, students received mind maps in different languages—one in Lithuanian and one in English. Students’ comments on the mind maps were recorded on a dictaphone.

2. Students’ answers to questions about the analysis of audiovisual material. After receiving the task sheets, the students first read and analyzed all the questions together with the researcher. Since there were an average of twelve questions (four questions for each level of Bloom’s taxonomy), the students answered the first six questions orally after the first viewing of the film and the remaining six after the second viewing. If necessary, the film was shown a third time. Some students wrote down their answers, but since the purpose of the task was to develop speaking skills, the students had to give their answers orally, thus supplementing their written notes. Questions for analyzing the visual materials (films) for all lessons are provided in Appendix B. Each student had a printed worksheet from Appendix B, which they filled out by hand. The children were introduced to the questions and tasks before watching the films and were able to answer the questions while watching the film.

3. Reflections. The reflection sheets helped to form a general impression of the students’ attitudes and emotions during and after the activity. Questions for reflection on all lessons are provided in Appendix C. Reflecting on their experiences, the students completed an open-ended questionnaire. Most of the questions were asked in Lithuanian, as the children do not yet have the necessary English language skills to reflect on their experiences in English.

4. Help system. During the lessons, students were provided with a help system developed by researchers called “Mr. Blue,” which they could use when they did not know certain words or the beginning of sentences while preparing answers to questions. The content of the help depended on the specific activity, the content of the film, the context, and what the students found most difficult. Appendix D presents the system of assistance for students.

#### 3.3.3. Stage 3—Analysis of Collected Data

The results of the study related to the development of students’ English and Lithuanian speaking skills using visual material analysis in two languages are highlighted. The empirical material was analyzed according to the cognitive speaking skills (knowledge and understanding, drawing conclusions, interpretation, and evaluation) that the children demonstrated when answering questions after viewing and analyzing visual material.

At this stage, specific data was selected and analyzed. We present the results of this analysis in Section 4. The responses of children who used the most words in English and non-repetitive responses in which students expressed their opinions and arguments were selected. The collected data was analyzed based on general Lithuanian and English language programs. In order to select the material for the analysis, all the collected data was examined: listening to the students’ answers, analyzing all the tasks performed by the students, analyzing the content of their reflections, and reviewing the notes taken during the study. The article presents only selected data that show the essence of the students’ answers. The data is presented according to the frequency of correct answers, with questions divided into groups covering cognitive abilities: knowledge and understanding, making conclusions, interpretation and evaluation.

### 3.4. Research Ethics

The research was conducted in a primary school with students aged 7–11. The research was carried out in three classes in accordance with the following mandatory ethical principles and requirements. Prior to the study, verbal consent was obtained from the school principal and the teachers of the third-grade classes. Written consent was obtained from the parents of the students participating in the study. Parents were assured that the results of the study would not be published with the personal data of specific children, that the identities of the students would not be disclosed, that confidentiality and anonymity would be maintained during the study, and that the data collected would be used only for scientific purposes. Before the study, the students were verbally informed about the study process. The study was conducted in such a way as not to disrupt the natural educational process. All data presented in the study are real, ensuring the principles of academic integrity. The study was approved by the university’s Didactics Research Cluster at its meeting in January 2024.

## 4. Analysis of Empirical Research Data

### 4.1. Lithuanian and English Language Lessons Analysis and Description of Context

Since the students’ speaking skills had rarely been developed prior to the study, (i.e.,), the students rarely had opportunities to speak, the authors of the study prepared preparatory activities in single-language lessons before integrating bilingual activities. During Lithuanian language lessons, children drew mind maps and discussed the topic *What does friendship mean to me?* (see Appendix A). All children who participated in the study expressed their thoughts about friendship in a sentence. Some of the participants (n = 12) were able to describe friendship using higher-level thinking (interpretation and evaluation) skills, saying that friendship for them is: *Friendship is when someone supports you, sympathizes with you, comforts you, and cheers you up when you are sad. Friendship is when someone helps you, advises you, supports you, and does not betray you. For me, friendship is support from friends, love, and sympathy. When I am having a hard time, a true friend helps me. When a friend helps you, when a friend listens to you, when a friend supports you*. After that, the children analyzed a recording of a conversation between the girl and her friend’s mother (the conversation was in Lithuanian). The analysis of the conversation covered aspects such as politeness and the girl’s behavior towards adults. Analysis of the responses to this task showed that all students were able to express their thoughts verbally, but their vocabulary levels differed. A small number of students (n = 8) described the girl’s behavior using several synonyms, for example, *The girl was polite, helpful, spoke kindly to her friend’s mother, and asked about her health*. The students were engaged and watched the film attentively. Since they had questions to analyze the film, they marked the answers as soon as they heard them (n = 36).

During the second Lithuanian language lesson, students analyzed and completed mind maps on the topic *Why is it important to resolve conflicts?* Some children (n = 12) were unable to discuss this issue even in Lithuanian, saying that they did not know why. Other children (n = 15) were able to discuss it in depth, demonstrating their interpretation and evaluation skills, stating that: *If we don’t resolve conflicts, there will be no friendship and no friendly people. It is important to resolve conflicts because your emotions and mental health depend on it. So that the world would not be gloomy, so that it would not be sad. So that you would have friends around you and would not always be alone. So that these conflicts would not lead to even bigger conflicts later on. Conflicts need to be resolved so that you can live and be friends.* After this part, the children listened to an audio recording of a Lithuanian fairy tale about the conflict between the sun and the moon, why one shines during the day and the other at night, and answered questions about the content of the fairy tale. All students who participated in the lesson (n = 40) answered questions related to knowledge and understanding directly related to the content of the fairy tale. The most difficult question was the one asking participants to describe the characters in the story using at least five synonyms. Only a small proportion of the children who took part in the study (n = 5) answered this question correctly.

During the English portion of the lesson, the children filled out a mind map by answering the question, *What is my favorite food?* In response to this question, the children repeated the names of food products or dishes in English, but most of the students’ answers were repetitive (n = 38), mentioning: *Pizza, milkshake, burger, orange, candy, salad, juice. Chicken, popcorn, fish, potato. Cheese, meat, water, apple.* After this activity, the children watched and analyzed the film *Pete the Cat’s Trip to the Supermarket.* Two questions about the content of the film, which asked children to list the characters (family members) in the film, were most often answered correctly (n = 38). The question “The family needs a lot of food. What do they do to remember* all the products?” highlighted the different abilities of the students in the class. 12 students answered this question correctly, and their answers were really detailed and comprehensive. For example, Domas said: *They made a list of all the food they needed*. (not only was this the correct answer, but he also used the past tense, which third-graders don’t usually know how to do yet). However, the question is considered to have been answered moderately well because other students were unable to answer in a complete sentence or were unable to formulate an answer at all. The question *Does dad like a hot dog? How do you know?* was answered completely and correctly by 10 students. The question Say at least one sentence* with the Present Simple tense, which was said in the film. Received only four correct answers. Although the question was discussed in detail with the researcher and the children are familiar with the grammatical tense, they still find it difficult to recognize it in speech. All children marked this question as the most difficult, even those who gave the correct answer.

At the beginning of the second English lesson, students filled out a mind map by answering the question *What names of emotions do I know?* (see Appendix A). Most students (n = 28) mentioned common, well-known emotions is *happy, sad.* There were students (n = 8) who demonstrated a deep understanding of emotions and used concepts such as: *angry, thirsty, cold, suspicious, disappointed, brave, disgusted, scared, excited, calm.* Later, the students analyzed an excerpt from the animated film *Inside Out* and answered questions about the film’s content orally. 42 students answered the question *What emotions can we see in the film?* correctly. All questions about the film are provided in Appendix B. All students participating in the study answered the question *What are the colors of the emotions in the film?* correctly. Both of these questions correspond to the level of knowledge on the cognitive scale. 6 students answered the question *What does Anger think about Riley’s classes?* correctly. This was a question that required watching the film for the third time and pausing at that particular point so that the children could hear clearly and specifically what was being said. Although this is a matter of interpretation, it was presented to children in an unfamiliar context. The 24 participants in the study identified the following issue of interpretation and assessment as the most difficult: *Imagine that you are the characters in the film, that it is your first day at school. How would you feel? Why?* It was difficult for children to empathize with another person’s role and identify that person’s emotions; only a very small proportion of the study participants (n = 5) answered this question correctly.

### 4.2. Analysis of the Results of Integrated Bilingual Lessons 1

During the activity, students watched the animated film *The Ant and The Grasshopper* in English, the content of which they had already analyzed in Lithuanian. It is important to note that the students performed these activities in pairs, as the bilingual education model is a new and untried method for them, and also so that the stronger students could help the weaker ones to get involved in the activity. The students were divided into pairs according to their English language skills. Each pair consisted of one student with a higher level of English and another with a basic or satisfactory level. The students began their analysis of the visual material by filling in a bilingual mind map (see Appendix A), which had the question *Why is work important?* (level of interpretation and evaluation) on the Lithuanian side where students had to provide reasoned answers, and on the English side, *Jobs* (level of knowledge and understanding), where students had to name known professions in English. The different levels of questions are due to the fact that children’s abilities in these two languages are different.

All students participating in the study (*N* = 46) completed the mind maps. However, 18 students, i.e., 9 pairs, provided very high-quality answers. Examples of students’ responses, based on the keywords in the mind maps, are presented in Table 4.

The answers given by the students in Table 4 show that the children’s English vocabulary related to professions corresponds to the content of the *Lithuanian General Program for The First Foreign Language* (2022). Although we have presented different answers given by children in the table, most of the pairs, after listening to their friends’ thoughts, not only added new words but also repeated those already heard from other children. The thoughts revealed by the students in the Lithuanian language mind map demonstrate that, already in the third grade, they have a broad understanding of the concept of work and are able to express meaningful arguments about why they consider work important and necessary.

After analyzing the mind maps on the work topic, the students watched a film about work and, after answering questions about their understanding of the content (see Appendix A), expressed their thoughts verbally. When preparing their answers to the questions, students could use a help system (see Appendix B). Table 5 presents the most common answers given by students to two questions in Lithuanian and two questions in English about the analysis of the audiovisual material.

We can say that the students answered questions directly related to the content of the animated film correctly. For example, all pairs of students (*N* = 46) who participated in the study gave correct answers to the questions *What did Grasshopper do in the summer?* and *What difficulties did Grasshopper encounter in the winter?* Although the wording of the answers varied slightly, the main idea was the same. For example, Orinta and Tadas said that *in the summer, Grasshopper played the violin*, while Arminas and Aistė said that *Grasshopper had fun*. Although both students’ answers are correct, one describes a specific action performed by a character in the work, while the other summarizes the character’s behavior by expressing his state of mind. This demonstrates the students’ higher-level thinking and verbal expression skills. In addition, after the activity, the students themselves assessed that the questions requiring knowledge and understanding, presented in Lithuanian, were the easiest.

A similar situation was observed with questions requiring knowledge and understanding, such as *Who remembered the Grasshopper in the winter?, What seasons can we see in the film?, What is the weather like in those seasons?,* which were presented to students in English. Most pairs of students (*n* = 38) answered these questions correctly, and many students rated them as the easiest questions in English.

Questions that developed interpretation and evaluation skills were difficult for students. For example, the question in Lithuanian, *How do you understand the word ‘morality’? Explain it in your own words or give a synonym*, was answered correctly by the smallest number of students (*n* = 8), who also rated it as the most difficult question (*n* = 40). In addition, students found it difficult to answer the question *What can we learn from this story? Say one sentence* in English, as most of them were unable to name what they had learned from the story (*n* = 40). Furthermore, all pairs of students (*n* = 46) participating in the study marked this question as the most difficult question in English. To answer this question, the information provided in the video is not enough; deeper reasoning skills are required, which students at the A1 level do not yet have. It is important to create conditions for developing such skills in the educational process, as these skills require a very broad vocabulary. Therefore, we can conclude that students are able to assess their work adequately—since the majority did not answer the question correctly, they indicated it as the most difficult.

### 4.3. Analysis of the Results of Integrated Bilingual Lessons 2

The integrated Lithuanian and English lessons differed from the lesson 2 in that the students watched the filmed material in Lithuanian, which was a short dialogue between children entitled *What are you doing during the holidays?* (level of making conclusions). The other essential principles of the lesson remained the same: students again worked in pairs consisting of members of different levels and answered questions in two languages. This time, the pairs were formed by other students so that they could try working with a different classmate. Students filled out a mind map *My activities on holidays* (level of knowledge and understanding) (see Appendix A).

All students (*N* = 46) completed the mind maps in pairs. The only difference was in the quality of the answers. Some students wrote only a few words on each map (filling in 2 or 3 columns). Other students wrote more (filling in 4 or 6 columns). Table 6 presents the main ideas expressed by the students based on the mind maps.

After analyzing the students’ thoughts, it was noticed that they were able to confidently express their ideas about their dream vacations in both English and Lithuanian. This is a particularly relevant and important topic for children, as they can communicate about it from their own experience. The students borrowed ideas from each other, what some listed in Lithuanian, others used in their answers in English. Although this was not recommended by the teacher, such examples show that by speaking two languages, students enrich their vocabulary in both languages and enrich each other when learning together.

After completing the mind map activity, the students watched a film and analyzed the visual material (see Appendix A). When answering the questions, students could also refer to the help system (see Appendix B). Table 7 provides examples of answers to the questions for analyzing the video.

In this lesson, students were most successful in answering questions (knowledge and understanding), such as *What does the boy do in the summer? Name at least two activities. What was the main topic of the children’s conversation?* All children (*N* = 46) correctly identified the boy’s activities, and all children also correctly identified the topic of the conversation. In addition, students marked these questions as the easiest questions in Lithuanian. The question in English, *Which kid (a boy or a girl) likes to draw?*, was answered correctly by all pairs of students (*N* = 46). All pairs (*N* = 46) also marked this question as the easiest question in English. Although it does not require much speaking, it requires careful watching of the film and listening to what the children are saying. The question *What school things can you see on the desk? Name at least two* was also answered by all pairs (*N* = 46). The students named more than two objects they saw. The question *Name all family members that the children mentioned* was easy because the children (*n* = 40) correctly named the family members in English and correctly identified which ones were mentioned by the boy and which ones by the girl.

The question *Why is the girl vacationing in the city?* was rated as moderately correct, as half of the students (*n* = 24) answered this question correctly. The question that was moderately fulfilled in English was *What do you like to do on holidays? Name two activities*, as less than half of the students (*n* = 20) answered it. The question *Which words show that the boy is happy about the upcoming vacation?* requiring interpretation and evaluation skills, was the most difficult to answer correctly, as only a small proportion of the students (*n* = 12) answered correctly. In addition, most pairs (N = 38) marked this question as the most difficult question in Lithuanian.

Both lessons, during which students analyzed visual material in two languages, Lithuanian and English, went smoothly for students who did not have such experience. They quickly grasped the lesson structure and, since they worked in pairs, helped each other and actively used the help system. It is important to note that even when faced with more difficult questions, students tried to answer verbally, drawing on the films they had watched, their own experience, or ideas from their friends’ answers. However, it was noted that students found it easier to answer questions in their dominant language (Lithuanian in our study), regardless of whether the visual material was in Lithuanian or English.

### 4.4. Analysis of Reflections

The students gave positive feedback on the content of the two language lessons (*n* = 42) because they were able to watch filmed material (*n* = 32) discuss it in pairs (*n* = 38), and answer questions orally (*n* = 30). Some students (*n* = 18) highlighted Mr. Blue’s help system, while some mentioned specific scenes from the film as their favorite aspects (*n* = 26). It was noted that some students mentioned the bilingual teaching method among the aspects they liked most. Here are some specific responses from students: *The lesson was interesting, you learn two languages at the same time, it’s much more fun and easier to answer in two languages* (Simona)*. It was more interesting to learn, it wasn’t difficult to answer in two languages when necessary, I liked having two languages in one lesson, it was fun to talk about the film in Lithuanian and English* (Domantas). The most difficult thing for the students was to answer questions in English. *It would have been better to have only one language because I didn’t understand everything in English* (Domantas). There were also children who said that they did not find anything difficult, so they had nothing to mention.

During the activity, students felt emotionally safe and motivated to learn. The reflections on both lessons were dominated by responses (*n* = 38) such as *good, very good, joyful, energetic*, etc. The students’ reasons for feeling this way were mostly related to their success in the lesson (answering most or all of the questions), and some students also pointed out that they felt good because they *worked in pairs* (*n* = 40). A few students who expressed negative feelings justified their answers by saying that they *were tired* or that *they preferred to analyze the film in one language*.

The arguments put forward by the students during the reflection were mainly related to the content of the tasks, but some children highlighted the advantage of learning both languages at the same time. Several children mentioned that they found this method easier. A small number of students (*n* = 2) said that they found it more convenient to learn in one language. It is likely that this response is related to the limited experience of children learning two languages at the same time.

In summary, it can be said that the reflections revealed the following attitudes among the students: watching and analyzing the film interested and engaged the students, many of them felt noticed and heard, and emphasized that they were able to express themselves (they lack this opportunity in other lessons).

## 5. Discussion and Conclusions

After analyzing the data from the empirical study, it can be stated that the bilingual audiovisual pedagogical intervention was implemented and observed. Students were successfully able to use their skills in both languages, demonstrating the efficacy of this pedagogical treatment (Castro et al., 2025; Ortiz et al., 2022). Visual material interests students, can be used as a tool to develop conversation, and encourages them to express their own experiences. Questions for analyzing visual material that encourage discussion and conversation develop students’ speaking skills, enable them to express their opinions and views, and develop their ability to perceive a film as a text. Our study confirmed the findings of other researchers (Fajardo Dack & Salamea Avila, 2023; Kathirvel & Hashim, 2020; Nair & Yunus, 2021) that audiovisual material helps develop students’ speaking skills and increases their motivation and self-confidence. In our study, the children’s responses were developed verbally in two languages, as the children enriched their English vocabulary and had the opportunity to express their opinions in Lithuanian.

During our study, performing activities in two languages not only improved the students’ English and Lithuanian language skills, but also developed their higher-order thinking skills, ability to adapt to changing tasks, and ability to express their opinions and experiences through the prism of two languages. However, we see room for discussion in the fact that the role of the teacher is important in the context of integrated bilingual learning, but it is not enough on its own. We fully agree with Beacco (2013) opinion, expressed more than a decade ago, that the issue of integrated didactics can be “alive” if there is unanimous agreement at all levels on the key factors determining the success of this idea: politics (Dixon, 2005; Mwaniki et al., 2017), ethics (Weinstock, 2022), professionalism (Gajo, 2006; Gajo & Grobet, 2011), responsibility, rights, and duties (Cummins, 2002). High-quality integrated language education is only possible when taking into account the dialogical nature of cultures, knowledge of them, and educational content that encompasses pragmatic and complex aspects, combining both global and local contexts. Indeed, our study confirms these insights, as the students, when answering questions in Lithuanian and English, participated in the educational process and drew on their knowledge to express their opinions and observations on various issues, i.e., they actively created content and used words and concepts learned in both languages (López-Pérez et al., 2023). The study showed that the bilingual education approach is flexible and suitable for the educational process, but whether its development will be long-term depends on education policy.

Based on the students’ views, we can conclude that the majority of students who participated in our study were in favor of a bilingual education model. This is evidenced by the following responses from students (*n* = 28): *the lesson was interesting, you learn two languages at the same time, it is easier to answer in two languages, it is fun to talk about the film in both Lithuanian and English*. After evaluating the answers revealed in the students’ reflections, we can conclude that the students felt that they had improved not only their speaking skills in Lithuanian, but also in foreign languages, and they also expressed a positive attitude towards the fact that they analyzed not only the text, but also visual material. In addition, we would like to emphasize the importance of the support system, which provided definitions of terms in both languages, because when developing bilingual education, it is important to express thoughts clearly and understand them adequately in both languages. This is confirmed by the research of other scholars (Gajo, 2006; Gajo & Grobet, 2011; Jakavonytė-Staškuvienė, 2017; Pantet, 2007), because tasks are particularly important from a didactic point of view, i.e., in terms of how knowledge, skills, and values are conveyed to learners, especially with regard to the formation and definition of terms and certain concepts. Bilingual education allows teachers to monitor students’ understanding and the growth of their abilities when children have to apply their language skills to solve real-life problems. It should be noted that concepts change depending on the subject taught, the project or the content of the activity, as this determines the specificity of the object being analyzed.

The choice of methods, which depends on the teacher’s pedagogical competence, has a significant impact on the development of students’ speaking skills (Zayas-Martínez et al., 2024). The development of speaking skills is facilitated by the verbal analysis of visual material, which can be developed in many different ways: through pair work, small group work, and individual presentations. Our research showed that working in purposefully formed pairs proved to be effective, as students were able to rely not only on visual material but also on each other’s experience. This is evidenced by the following responses from students (N = 26): *I learned many new words; when I didn’t know the answers, I could ask my classmate for advice, and he helped me; it was interesting to watch the film in one language and answer questions in another; we prepared the answers together, discussing them*. We used mind maps that have already been scientifically validated by other researchers (Brohy, 2002; Jakavonytė-Staškuvienė, 2017; Kirckhoff, 1992; Krekeler, 1997). Indeed, the use of mind maps in two languages as an aid to preparing for conversation proved very effective in our study, demonstrating the suitability of this method for the current educational context. Some children (N = 5) said that it was difficult to analyze the film in English because they did not understand everything. Here are some examples of their responses: *It was easier to answer questions in Lithuanian, but it was difficult to complete the tasks in English because I did not understand everything*. In addition, some children (*n* = 18) noticed the advantage of the support system because they were able to participate more actively in the lesson: *Mr. Blue helped me; using Mr. Blue’s words, I was able to talk about myself in English. I like English, especially when I can speak it more and learn new words*. It is important to note that student engagement was significantly higher than in regular lessons. This means that the bilingual education experience interested the children, and they used new words in both English and Lithuanian.

## 6. Limitations of the Study

All data collected during the study are authentic and meaningful, as they reveal the development of students’ speaking skills through the use of innovative teaching methods that combine the learning of two languages. However, as the study was conducted in one specific school, it does not show the full range of students’ speaking skills, so when planning such activities with other students, the results may differ as they will depend on the context and the students’ experience. It is also important to note that although the study only involved third-grade students, it reveals certain trends and student experiences and provides tools that could be used by researchers and teachers in other countries in similar studies. In order to apply the research data to a wider sample, a similar study should be conducted in several different places of residence, including small towns and villages. In our case, the study was conducted in one of Lithuania’s major cities. If the study had been conducted with a larger number of students, its data could have been applied to the entire population.

Another limitation of the study is its duration. If similar educational activities were carried out over a longer period of time, it is likely that both the results of the students’ activities and their responses after the activities would reflect their greater experience.

Our study was developed in accordance with the parameters of qualitative research. Of course, when conducting similar studies with a wider scope and a larger sample size, it would be possible to apply an experimental design, use data comparison and integrate standardized measures of language proficiency in a pre-test/post-test design.

## Figures and Tables

**Table 1:** Abstract concepts that should be learned by students in grades 3–4 (according to the document *Lithuanian General Program for the First Foreign Language*, 2022).

Abstract Concept	Contents
Existence	Being, non-being as existence. Being, non-being in a specific place. Having, not having. Happening, non-happening.
Area	Location. Layout. Distance.
Movement	Method. Direction. Origin by location. Distribution. Speed.
Dimensions, parameters	Size. Shape. Length, width. Area. Weight. Temperature.
Time	Specific and/or defined time. Time reference. General classification. Repetition. Anticipation, recurrence. Delay. Sequence. Reference to the present. Reference to the past. Reference to the future. Continuous duration. Interrupted action. Continuity. Temporariness. Beginning. End. Constancy. Change.
Quantity	Number. Quantity. Degree. Comparison.
Physical characteristic	Dryness, moisture. Visibility and/or appearance. Audibility and/or sound. Taste. Smell. Texture and/or texture. Color. Age. Physical condition (of a person or object). Accessibility. Cleanliness. Material. Fullness, emptiness.
Evaluated characteristic	Value, price. Quality. Fairness, unfairness. Success, failure. Ability, inability. Importance, unimportance. Difficulty, ease.
Connections and relationships	Relationships between actions and/or events. Instrument, means, method. Recipient. Dependence and/or ownership. Connection. Inclusion, separation, exclusion. Cause, motive. Purpose. Definiteness, indefiniteness.

**Table 2:** Expression of Lithuanian language speaking skills among 3rd and 4th grade students (according to the document *Lithuanian Language and Literature General Program for Primary Education*, 2022).

Achievements	Learning Content for Grades 3–4
A2. Participates in various communication situations, listens and speaks, taking into account the situation, purpose, and audience	Preparation for speaking a monolog (independently, purposefully, in a planned manner), participation in conversations, inclusion of nonverbal means of communication. Consideration of the addressee and the communication situation—demonstrating interest, respecting opinions, observing language etiquette. Emotional, expressive, self-expressive and engaging storytelling, coherent speech, development of the topic. Detailed description, clear information, explanation of what is being done or what happened and how. Expressing and justifying one’s opinion, relying on personal experience and known context.
A3. Uses linguistic means appropriately and adheres to language norms	Correct pronunciation, stress, intonation, pausing, and logical stress. Also, enriching language with new words and expressions and applying them in different situations.
A4. Uses listening and speaking strategies	Reflecting on one’s own speech, explaining one’s successes and failures, justifying and suggesting what could be done better (this skill is developed with the help of the teacher). Experimenting with and applying various speaking strategies or strategies for preparing for speech.

**Table 3:** Film analysis in general primary education programs for Lithuanian language and literature and first foreign language (2022).

Film Analysis in the General Program of the Lithuanian Language and Literature	Film Analysis in the First Foreign Language General Program
Cognitive skills: interpretation is the students’ comments on and reveals their own interpretation of the meaning of the film. Students develop their civic identity and civic power by evaluating the content of audiovisual texts. Cultural expression skills: works are presented that feature interesting expressive solutions (e.g., films, videos). Links to literature and other arts: films based on literary works are discussed. Selected periodicals for children (e.g., films) are discussed.	Students develop digital competence when they understand the videos and films they watch. They understand the theme of all short, simple videos and animated films about familiar topics. A separate skill is identified—the comprehension of audiovisual text.

**Table 4:** Students’ thoughts about work and professions, expressed verbally.

Students	Answers in Lithuanian (Level of Interpretation and Evaluation)	Answers in English (Level of Knowledge and Understanding)
Arminas and Aistė	*Kad* *sumokėtum mokesčius, paskolas, kad nusipirktum valgyti [To pay taxes, loans, to buy food].*	*Policeman, doctor, teacher, actor.*
Orinta and Tadas	*Kad* *uždirbtum pinigų, galėtum išsilavinti ir pirkti tai, ko nori [To earn money, get an education, and buy what you want].*	*Zookeeper, singer, nurse, housewife.*
Edita and Laura	*Kad* *būtum atsakingas, turėtum pinigų ir kad galėtum ko nors naujo išmokti [To be responsible, have money, and be able to learn something new].*	*Housewife, fireman, driver, designer.*

Note: The table shows the exact answers given by students during the study, marked in italics.

**Table 5:** Examples of students’ answers to questions in an integrated Lithuanian and English lesson, revealing the speaking skills of students at different levels (Lesson 1).

(a) Apibūdink Skruzdės būdą bent dviem žodžiais. [Describe the Ant’s behavior in at least two words.] (level of interpretation and evaluation) (*n* = 20) (b) Palygink, kuo Žiogo būdas skiriasi nuo Skruzdės. [Compare how the way a Grasshopper walks differs from that of an Ant.] (level of interpretation and evaluation) (*n* = 20)		
**Students**	**Students’ answers**	**Researchers’ comments**
Simona and Aistis	*(a) Meili, nes padėjo Žiogui, ir darbšti, nes dirbo visą vasarą [She was kind because she helped the Grasshopper, and she was hardworking because she worked all summer].* *(b) Skruzdė buvo darbšti ir geraširdė, o Žiogas buvo tinginys ir visai nedarbštus [The Ant was hardworking and kind-hearted, while the Grasshopper was lazy and not hardworking at all].*	After analyzing the answers, it can be seen that the students know various synonyms *good, kind, warm-hearted, friendly, sincere*. The students perfectly link the film to the question asked and give specific, accurate answers.
Orinta and Tadas	*(a) Draugiška ir gera. Ji davė Žiogui žiemą valgyti [Friendly and kind. She gave Grasshopper food to eat in winter].* *(b) Skruzdė dirbo, kaupė atsargas, o Žiogas nedirbo, nekaupė maisto žiemai [The Ant worked and gathered supplies, while Grasshopper did not work and did not gather food for winter].*	
Domas and Tomas	*(a) Geraširdė ir darbšti, nes Žiogas jos neklausė vasarą, bet ji jam vis tiek padėjo žiemą [She is kind-hearted and hard-working because the Grasshopper did not listen to her in the summer, but she still helped him in the winter].* *(b) Žiogas tinginys ir užsispyręs, Skruzdė darbšti ir nuoširdi [The Grasshopper is lazy and stubborn, while the Ant is hard-working and sincere].*	
Įsivaizduok, jog esi Skruzdė. Ar priimtum Žiogą žiemą, net jei jis nepaklausė tavo patarimo? Atsakymą argumentuok. [Imagine you are an Ant. Would you take in a Grasshopper in winter, even if he didn’t ask for your help? Explain your answer.] (level of interpretation and evaluation) (*n* = 18)		
Arminas and Aistė	*Taip, mums* *būtų jo gaila žiemą, todėl priimtume ir pasakytume, jog reikia rūpintis savimi [Yes, we would feel sorry for him in winter, so we would accept and say that he needs to take care of himself].*	The students discussed this question at length and debated how they would act. The answers given by each pair of students were very different, revealing their moral attitudes, beliefs, ability to argue coherently and empathize with another character (in this case, the Ant).
Edita and Laura	*Taip,* *nes jis buvo išalkęs ir sušalęs ir labai prašėsi užeiti į namus [Yes, because he was hungry and cold and begged to come in].*	
Aurelija and Domantas	*Ne,* *nes jis buvo tinginys ir neklausė patarimo [No, because he was lazy and didn’t listen to advice].*	
What is the Ant doing in the summer? (level of knowledge and understanding) (*n* = 42)		
Orinta and Tadas	*The ant is working to have food.*	Since the question concerns knowledge and understanding, the students’ answers are similar, but it can be noted that each pair presents the answer from a different perspective, i.e., they work to have food, they store food for winter, they work hard in summer. The students structure their sentences correctly, and it has also been noted that they use words from the film to expand the content of their sentences.
Domas and Tomas	*The ant is gathering food for the winter.*	
Aurelija and Domantas	*The ant is working hard in the summer.*	
Give advice to the Grasshopper to make his winter easier. (level of making conclusions) (*n* = 30)		
Edita and Laura	*Build a house in the summer because it is cold in winter.*	This question was answered most effectively—all pairs of students gave the Grasshopper lots of advice. The students based their advice on the film and their own knowledge, and helped each other with words they knew. Also, from Simona and Aistis’ answers, it can be seen that the pair relied on the support system.
Arminas and Aistė	*Start working, because job is first, fun is later.*	
Simona and Aistis	*My advice for the grasshopper would be: don‘t be lazy and go to work.*	

Note: The table shows the exact answers given by students during the study, marked in italics.

**Table 6:** Examples of students’ thoughts about holidays, expressed verbally.

Students	Answers in Lithuanian (Level of Making Conclusions)	Answers in English (Level of Knowledge and Understanding)
Simona and Aistė	*Saulė,* *jūra, pliažas ir geros draugės [Sun, sea, beach, and good friends].*	*Riding a bike, playing with friends, swimming, sunbathing, eating watermelon and no school.*
Tadas and Arminas	*Geras* *oras, ledai, krepšinis ir laikas su draugais [Good weather, ice cream, basketball, and time with friends].*	*Basketball, football, tennis, sea, computer games, playground,*
Aurelija and Aistis	*Žaidimai su draugais, kelionės, važiuoti pas senelius, važinėtis dviračiu [Playing games with friends, traveling, visiting grandparents, riding a bike].*	*Sleep, be with friends, playing games, listen to music, visit grandma and grandpa, read a book.*

Note: The table shows the exact answers given by students during the study, marked in italics.

**Table 7:** Examples of students’ answers to questions in an integrated Lithuanian and English lesson, revealing the speaking skills of students at different levels (Lesson 2).

Berniukas pasakė: „Per atostogas visada smagu”. Kaip per atostogas jautiesi tu? Kodėl? [The boy said, “Holidays are always fun.” How do you feel during the holidays? Why?] (level of interpretation and evaluation) (*n* = 24)		
**Students**	**Students’ answers**	**Researchers’ comments**
Arminas and Tadas	*Gerai* *, nes galime kasdien žaisti futbolą ir krepšinį ir nereikia anksti keltis [That’s good because we can play soccer and basketball every day and don’t have to get up early].*	When analyzing the students’ responses, it was noticed that children often described their well-being using the word “good,” which indicates that children do not know the words to describe their feelings. It is positive that children came up with individual reasons why they feel “good.” Also, many pairs of students said that they felt good *because they could do something*, but did not specify what.
Orinta and Domantas	*Linksmai, nes galima aplankyti įvairių pasaulio vietų ir sužinoti vis kažką arba su artimiausiais draugais ir šeima vykti prie ežero su palapinėm [It’s fun because you can visit different places around the world and learn something new, or go to the lake with your closest friends and family and pitch a tent].*	
Simona and Aistė	*Gerai, nes galima aplankyti žmones, kuriuos seniai matei ir aktyviai leisti laiką, ten pavyzdžiui, su dviračiais [It is beneficial because you can visit people you have not seen for a long time and actively spend time there, for example, riding bicycles].*	
Prisimink savo praėjusios vasaros nuotykius. Kuris iš jų buvo įsimintiniausias ir kodėl? Bent dvejais sakiniais papasakok apie jį. [Think back to your adventures last summer. Which one was the most memorable and why? Describe it in at least two sentences.] (level of interpretation and evaluation) (*n* = 20)		
Edita and Tomas	*Edita: kai per praeitas vasaros atostogas pirmą kartą skridau lėktuvu. Man labai patiko, nes nuo šešių metų svajojau kažkur skristi ir mano svajonė išsipildė [when I flew on an airplane for the first time last summer. I really enjoyed it because I had dreamed of flying somewhere since I was six years old, and my dream came true].* *Tomas: kai laimėjau futbolo varžybas. Tos varžybos tokios buvo svarbios, daug ruošiausi, ir laimėjau [when I won the soccer game. That game was so important, I prepared a lot, and I won].*	This question was discussed most extensively by all students, who listened very attentively to each other and responded to their friends’ stories. Although the students worked in pairs, they were asked to think about this question independently and individually. This was performed so that all students could verbally express what was memorable to them and what was personally important to them. All the stories were very different, and the students tried to answer in two sentences, but after sharing, they had questions for each other that would have been unfair not to ask. Therefore, the lesson lasted longer than planned. It is important to note that if the topic is relevant and interesting to children, and they are able to express their experiences more freely, then students speak boldly, share their experiences, and even want to continue the discussion after the lesson has ended. Such situations demonstrate the quality of the educational process and the possibilities for involving every child.
Domas and Laura	*Domas: kai po ilgo laiko aš susitikau su savo geru draugu, kuris gyvena Vokietijoje. Ta diena buvo geriausia vasaros diena, nors įsimintinų įvykių buvo daug [when, after a long time, I met my good friend who lives in Germany. That day was the best day of the summer, even though there were many memorable events].* *Laura: įsimintiniausias nuotykis man kai aš suradau mažą, vienišą kačiuką ir jisai buvo iki rugsėjo mėnesio pas mus. Tada mes radom jam šeimininkus [my most memorable adventure was when I found a small, lonely kitten, and he stayed with us until September. Then we found him a home].*	
Aurelija and Aistis	*Aurelija: aš per vasaros atostogas buvau laukiniam paplūdimyje Latvijoje. Šis įvykis man įsimintiniausias, nes laukiau jo visą vasarą ir ten buvo labai gražu [during my summer vacation, I went to a wild beach in Latvia. This event was the most memorable for me because I had been looking forward to it all summer, and it was very beautiful there].* *Aistis: mano nuotykis liūdnas, kai nukritau nuo dviračio. Aš tada susilaužiau koją ir gulėjau visą vasarą namie [my adventure was sad when I fell off my bike. I broke my leg and had to stay home all summer].*	
Where are the children? How do you know? (level of making conclusions) (*n* = 46)		
Arminas and Tadas	*They are in school because there are markers, papers, books on the table.*	This question revealed the different things that students notice and the reasons why they decided that children are at school. The children supported their answers with arguments and demonstrated a wide vocabulary related to school items.
Domas and Laura	*In school because of school items like pencils, pens, study books and everything else.*	
Orinta and Domantas	*Children are in the classroom because kids wearing school uniforms.*	
The girl mentioned that she goes to the playground on holidays. Do you go to the playground? When and with whom? (level of making conclusions) (*n* = 30)		
Aurelija and Aistis	*Yes, we go to the playground with our family in the summer.*	The students identified this question as the difficult one to answer in English, but it was unique in that the students answered it in parts: first, they answered each question separately, and then combined their answers into a single sentence. In addition, the students actively used the help system when answering this question.
Simona and Aistė	*I go to the playground with my friends in the summer.*	
Edita and Tomas	*Sometimes with friends in summer.*	

Note: The table shows the exact answers given by students during the study, marked in italics.

## Data Availability

Data is contained within the article.

## Appendix A. Mind Maps

Mind map for 1st Lithuanian language lessons, using program Canva Pro
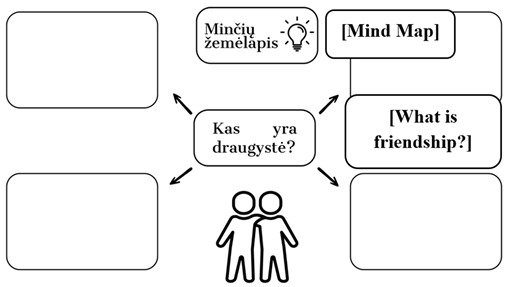
Mind map of the 2nd Lithuanian language lessons, using program Canva Pro
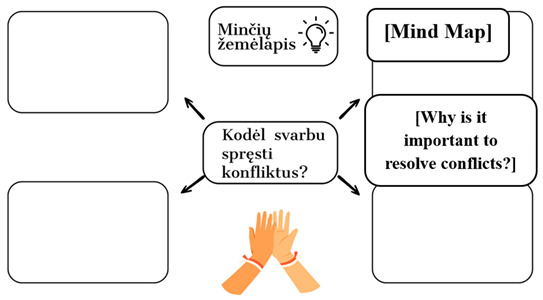
Mind map of the 1st English lesson, using program Canva Pro
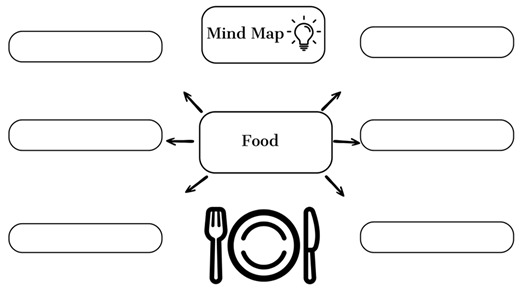
Mind map of the 2nd English lesson, using program Canva Pro
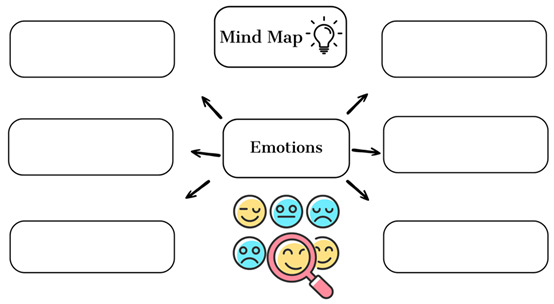
Mind maps 1st integrated bilingual (Lithuanian and English) lesson, using program Canva Pro
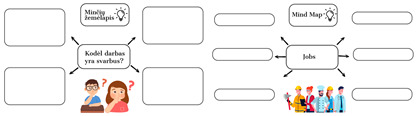
Mind maps 2nd integrated bilingual (Lithuanian and English) lesson, using program Canva Pro
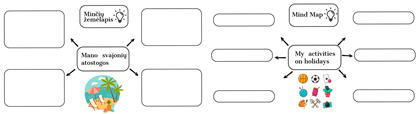

## Appendix B. Visual Material Analysis Tasks

1st Lithuanian language lesson visual material analysis tasks, using program Canva Pro
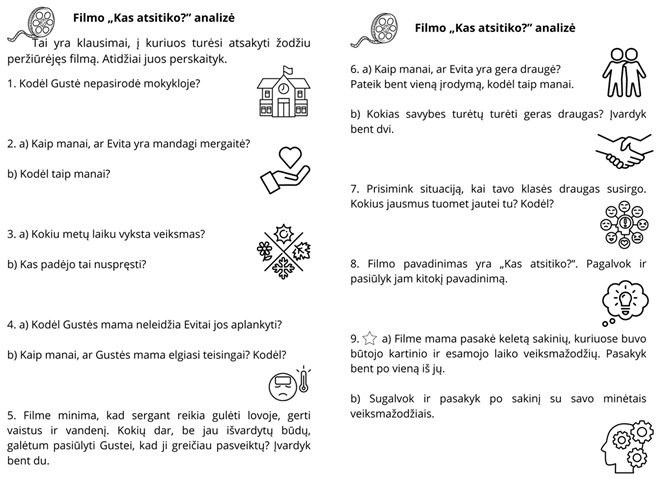
2nd Lithuanian language lesson visual material analysis tasks, using program Canva Pro
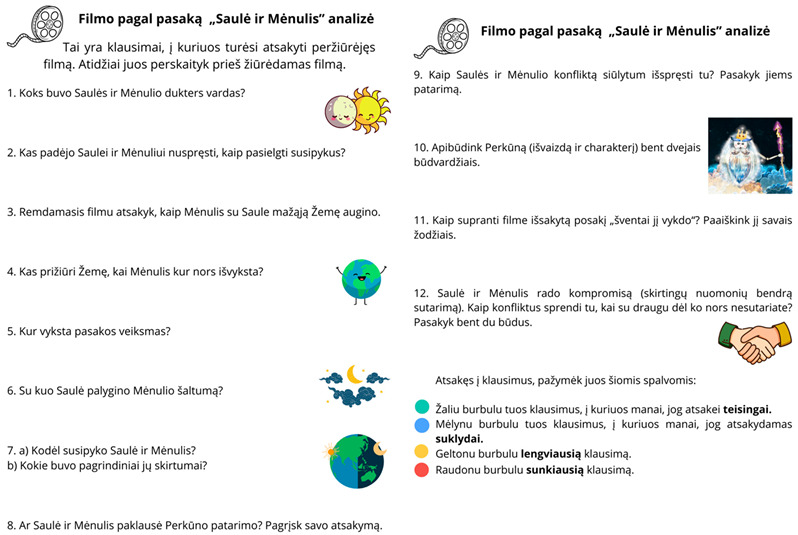
1st English lesson visual material analysis tasks, using program Canva Pro
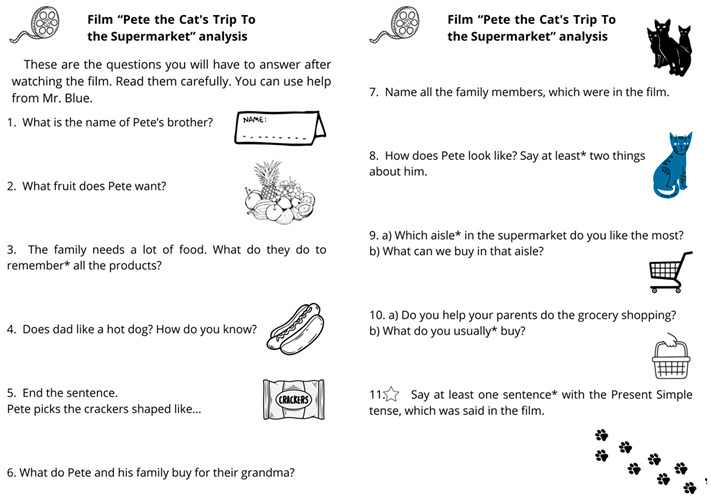
2nd English lesson visual material analysis tasks, using program Canva Pro
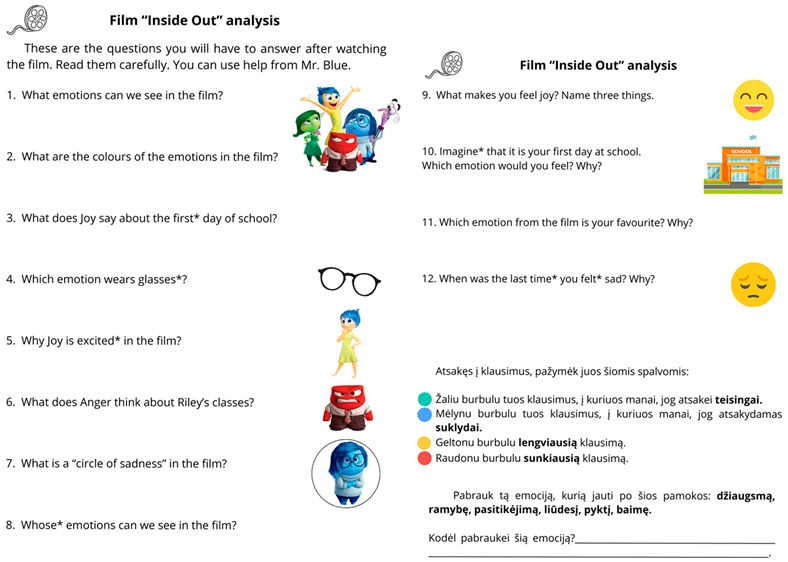
Example of integrated bilingual (Lithuanian and English) audiovisual material tasks for Lesson 1 (prepared by the authors of this paper using program Canva Pro)
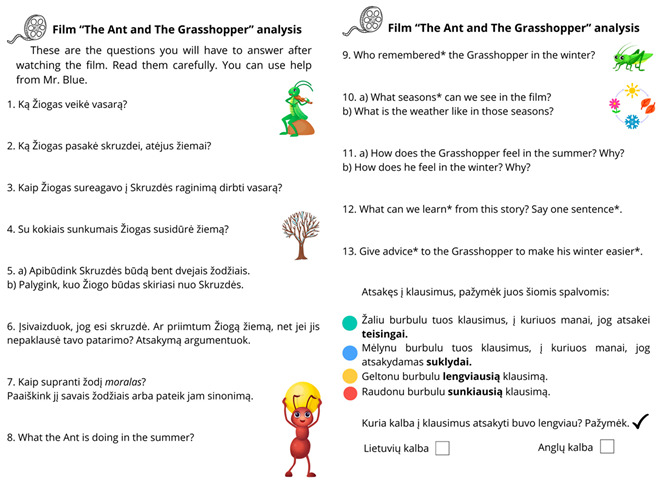
Example of integrated bilingual (Lithuanian and English) audiovisual material tasks for Lesson 2 (prepared by the authors of this paper using program Canva Pro)
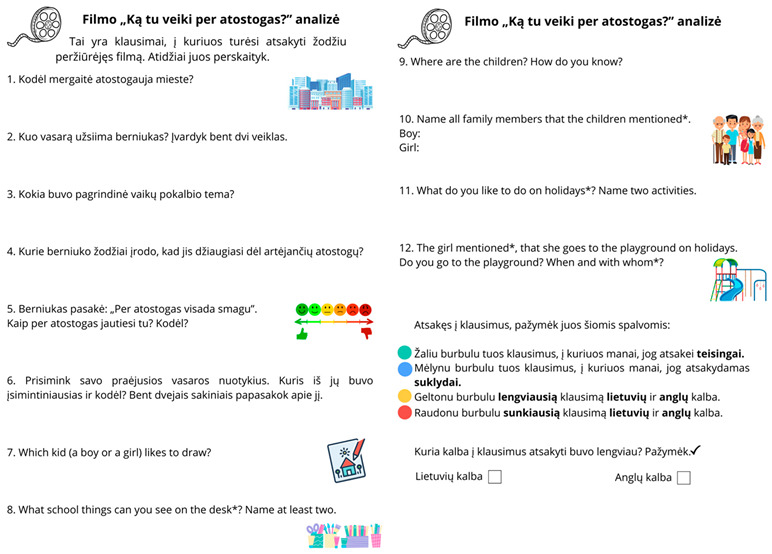

## Appendix C. Reflections Using the Unfinished Sentence Method, Applied After Educational Activities

Reflections on Lithuanian language lessons
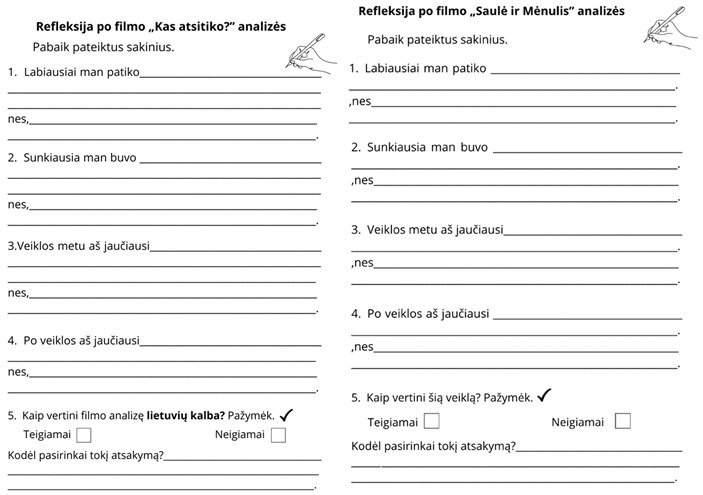
Reflections on English lessons
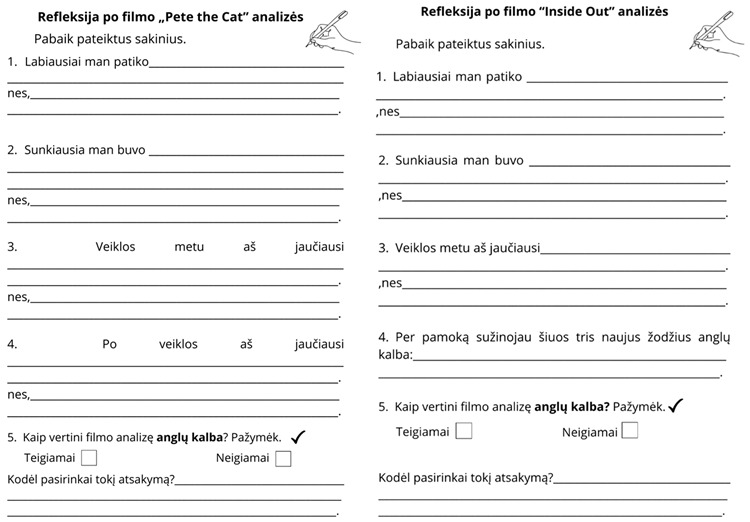
Reflections on integrated Lithuanian and English language lessons
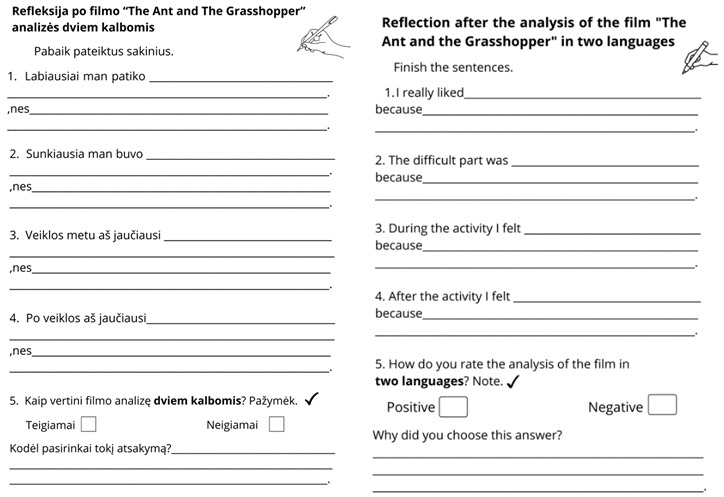

## Appendix D. Mr. Blue Support System

Support systems for English language lessons, using program Canva Pro
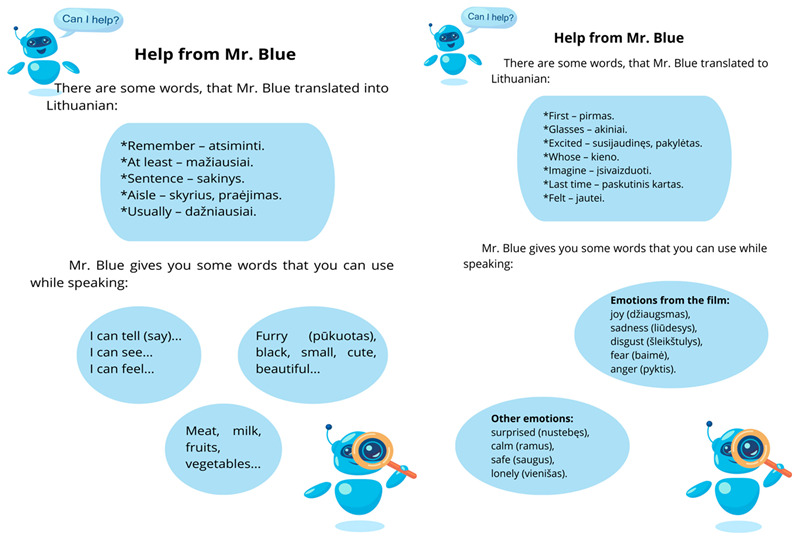
Help system for integrated Lithuanian and English language lessons, using program Canva Pro

## References

[B1-behavsci-15-01362] Ahmad S. (2015). Improving students skills in translations by using students-teams achievement division (STAD) technique. Al-Ta’lim.

[B2-behavsci-15-01362] Ahmad Z. (2020). Peer observation as a professional development intervention in EFL pedagogy: A case of a reading lesson on developing the top-down processing skills of the preparatory year students. Online Submission.

[B3-behavsci-15-01362] Aksu Ataç B., Günay-Köprülü S. (2018). The role of subtitles in foreign language teaching. International Online Journal of Education and Teaching.

[B4-behavsci-15-01362] Aljohani M. (2017). Principles of “constructivism” in foreign language teaching. Journal of Literature and Art Studies.

[B5-behavsci-15-01362] Alluri P. (2018). Enhancing English language teaching through films in general foundation programs. Arab World English Journal.

[B6-behavsci-15-01362] Ampa A. T., Rasyid M. A., Rahman M. A., Haryanto H., Muhammad Basri D. (2013). The implementation of multimedia learning materials in teaching English speaking skills. International Journal of English Language Education.

[B7-behavsci-15-01362] Anderson L. W., Krathwohl D. R. (2001). A taxonomy for learning, teaching and assessing: A revision of bloom’s taxonomy of educational objectives: Complete edition.

[B8-behavsci-15-01362] Antar D. (2023). The use of drama in developing the skill of speaking in standard Arabic among third grade Arabic speaking students. Theory & Practice in Language Studies (TPLS).

[B9-behavsci-15-01362] Ashcroft R. J., Garner J., Hadingham O. (2018). Incidental vocabulary learning through watching movies. Australian Journal of Applied Linguistics.

[B10-behavsci-15-01362] Avello D., Muñoz C. (2023). The development of receptive language skills from captioned video viewing in primary school EFL learners. Education Sciences.

[B11-behavsci-15-01362] Baba K. (2009). Aspects of lexical proficiency in writing summaries in a foreign language. Journal of Second Language Writing.

[B12-behavsci-15-01362] Beacco J.-C. (2013). Éthique et politique en didactique des langues. Autour de la notion de responsabilité.

[B13-behavsci-15-01362] Biglari N., Struys E. (2021). Native language interference in English L2 word recognition and word integration skills. Theory & Practice in Language Studies (TPLS).

[B14-behavsci-15-01362] Bloom B. S., Engelhart M. D., Furst E. J., Hill W. H., Krathwohl D. R. (1956). Taxonomy of educational objectives: The classification of educational goals. Vol. Handbook I: Cognitive domain.

[B15-behavsci-15-01362] Bottomley J. (2018). Talking movies: Using film as part of language study and academic development. The Journal of Research and Scholarly Activity at University Campus Oldham.

[B16-behavsci-15-01362] Brohy C. (2002). L’enseignement bilingue dans/par/à travers la forêt = Zweisprachiger Unterricht im/mit dem Wald und durch den Wald: Actes des 4^èmes^ Rencontres des enseignant(e)s bilingues.

[B17-behavsci-15-01362] Carignan I., Beaudry M.-C., Larose F. (2016). La recherche-action et la recherche-développement au service de la littératie.

[B18-behavsci-15-01362] Castro D. C., Franco-Jenkins X., Chaparro-Moreno L. J. (2025). The effects of dual language education on young bilingual children’s learning: A systematic review of research. Education Sciences.

[B19-behavsci-15-01362] Cicėnaitė-Milaševičiūtė A. D., Juškevičienė A. (2020). Pradinių klasių mokinių kalbinių gebėjimų ugdymas(is) taikant neurodidaktikos principus lietuvių kalbos pamokose [Development of primary school student language skills: Application of principles of neurodidactics in Lithuanian language classes]. Švietimas: Politika, vadyba, kokybė/Education Policy, Management and Quality.

[B20-behavsci-15-01362] Cummins J. (2002). Rights and responsibilities of educators of bilingual/bicultural children. Counterpoints. Making a Difference in the Lives of Bilingual/Bicultural Children.

[B21-behavsci-15-01362] Davis L. (2023). Ideological foundations, curricular models, and the path of bilingual education. Curriculum & Teaching Dialogue.

[B22-behavsci-15-01362] Dixon L. Q. (2005). Bilingual education policy in Singapore: An analysis of its sociohistorical roots and current academic outcomes. International Journal of Bilingual Education and Bilingualism.

[B23-behavsci-15-01362] Djamàa S. (2018). From book to screen: Adopting cinematic adaptations of literature in the EFL classroom to hone students’ critical thinking skills. Computers in the Schools.

[B24-behavsci-15-01362] Eden C., Ackermann F. (2018). Theory into practice, practice to theory: Action research in method development. European Journal of Operational Research.

[B25-behavsci-15-01362] Elt O. U. P. (2015). Mother Language Day: Why learning a foreign language is important.

[B26-behavsci-15-01362] Ergül H. (2023). Translanguaging realities: The use of first language in microteaching practices vs. young learner classrooms. Bartin University Journal of Faculty of Education.

[B27-behavsci-15-01362] Fajardo Dack T. M., Salamea Avila M. J. (2023). Developing speaking skills in EFL young learners through visual and audiovisual materials. Revista Metropolitana de Ciencias Aplicadas.

[B28-behavsci-15-01362] Fu X., Relyea J. E. (2024). Exploring the role of mind mapping tools in scaffolding narrative writing in English for middle-school EFL students. Education Sciences.

[B29-behavsci-15-01362] Gajo L. (2006). Types de savoirs dans l’enseignement bilingue: Problématicité, opacité, densité. Education et Sociétés Plurilingues.

[B30-behavsci-15-01362] Gajo L., Grobet A., Berthaud A.-C., Gradoux X., Steffen G. (2011). Saturation des savoirs et variété des enseignements bilingues. Plurilinguismes et construction des savoirs. Cahiers de l’ILSL.

[B31-behavsci-15-01362] Güneş Ç., Sarıgöz İ. H. (2021). Speaking struggles of young EFL learners. International Journal of Curriculum and Instruction.

[B32-behavsci-15-01362] Hestiana M., Anita A. (2022). The role of movie subtitles to improve students’ vocabulary. Journal of English Language Teaching and Learning.

[B33-behavsci-15-01362] Jakavonytė-Staškuvienė D. (2017). Kalbų didaktikos integravimas kitų dalykų pamokose pradinėse klasėse (Šveicarijos patirtis) [Integration of language teaching into other subjects in primary school (Swiss experience)]. Towards research-based education: An edited collection of research studies.

[B34-behavsci-15-01362] Jakavonytė-Staškuvienė D., Žemgulienė A. (2016). Pradinių klasių mokinių kalbinių gebėjimų integruotas ugdymas įvairių dalykų pamokose: Istorinio laiko tėkmės supratimo atvejis [Integrated development of language skills of primary school pupils in various subjects: The case of understanding the historical timeline]. Verbum.

[B35-behavsci-15-01362] Jayakodi K., Bandara M., Perera I., Meedeniya D. (2016). WordNet and cosine similarity based classifier of exam questions using bloom’s taxonomy: International journal of emerging technologies in learning. International Journal of Emerging Technologies in Learning.

[B36-behavsci-15-01362] Kathirvel K., Hashim H. (2020). The use of audio-visual materials as strategies to enhance speaking skills among ESL young learners. Creative Education.

[B37-behavsci-15-01362] *Key Competences for Lifelong Learning in the European Schools* (2018). Schola Europaea: Office of the Secretary-General, Pedagogical Development Unit. https://www.eursc.eu/BasicTexts/2018-09-D-69-en-2.pdf.

[B38-behavsci-15-01362] Kirckhoff M. (1992). Mind mapping. Einführung in eine creative methode.

[B39-behavsci-15-01362] Kiruthiga E., Christopher G. (2022). The impact of affective factors in English speaking skills. Theory & Practice in Language Studies.

[B40-behavsci-15-01362] Krekeler C. (1997). Mind Mapping im Unterricht. Materialien Deutsch ALS Fremdsprache, Heft.

[B41-behavsci-15-01362] Kurt D. S. (2021). Constructivist learning theory. Educational Technology.

[B42-behavsci-15-01362] Lee S. H., Muncie J. (2006). From receptive to productive: Improving ESL learners’ use of vocabulary in a post-reading composition task. TESOL Quarterly.

[B43-behavsci-15-01362] Li Y. (2025). Listen or read? The impact of proficiency and visual complexity on learners’ reliance on captions. Behavioral Sciences.

[B44-behavsci-15-01362] *Lithuanian General Program for The First Foreign Language* (2022). Lietuvos bendroji pirmosios užsienio kalbos programa [Lithuanian general program for the first foreign language].

[B45-behavsci-15-01362] *Lithuanian Language and Literature General Program for Primary Education* (2022). Lietuvos pradinio ugdymo lietuvių kalbos ir literatūros bendroji programa [Lithuanian language and literature general program for primary education].

[B46-behavsci-15-01362] Lo H.-C., Wang T.-H. (2024). A study on the design of embedded visual image teaching aids to assist young children’s cognitive and fine motor development. Journal of Intelligence.

[B47-behavsci-15-01362] López-Pérez M., de la Montaña Conchiña J. L., Bravo Galán J. L., de la Maya Retamar G. (2023). Bilingual science lexicon of pre-serviced primary school teachers. Education Sciences.

[B48-behavsci-15-01362] Mat Halif M., Hassan N., Sumardi N. A., Shekh Omar A., Ali S., Abdul Aziz R., Abdul Majid A., Salleh N. F. (2020). Moderating effects of student motivation on the relationship between learning styles and student engagement. Asian Journal of University Education.

[B49-behavsci-15-01362] Mereckaitė I. (2022). Language as a tool empowering students to be independent in sociocultural environment. Journal of Education, Culture & Society.

[B50-behavsci-15-01362] Mwaniki M., Arias M. B., Wiley T. G., García O., Lin A., May S. (2017). Bilingual education policy. Bilingual and multilingual education. Encyclopedia of language and education.

[B51-behavsci-15-01362] Nair V., Yunus M. M. (2021). A systematic review of digital storytelling in improving speaking skills. Sustainability.

[B52-behavsci-15-01362] Oh M. H., Mancilla-Martinez J., Hwang J. K. (2023). Revisiting the traditional conceptualizations of vocabulary knowledge as predictors of dual language learners’ English reading achievement in a new destination state. Applied Psycholinguistics.

[B53-behavsci-15-01362] Olinghouse N. G., Wilson J. (2013). The relationship between vocabulary and writing quality in three genres. Reading and Writing.

[B54-behavsci-15-01362] Ortiz A. A., Fránquiz M. E., Lara G. P. (2022). Educational equity for emergent bilinguals: What’s wrong with this picture?. Bilingual Research Journal.

[B55-behavsci-15-01362] Pantet J. (2007). Ces obscurs objets de l’acquisition: Objets traités et modes de traitement dans l’acquisition du Français Langue Etrangère.

[B56-behavsci-15-01362] Petrošienė K. (2022). Mąstytojų ugdymas Lietuvoje: Sekant J. Baranovos ir L. Duoblienės kelrodžiu [The education of thinkers in Lithuania: Following the example of J. Baranova and L. Duoblienė]. Problemos.

[B57-behavsci-15-01362] Qi W., Jiang Y. (2021). Use of a Graphic Organiser as a pedagogical instrument for the sustainable development of EFL learners’ English reading comprehension. Sustainability.

[B58-behavsci-15-01362] Rakickienė L. (2021). Mokyklinio amžiaus vaiko ir jaunuolio kognityviosios ir socialinės-emocinės raidos aprašas [Description of the cognitive and social-emotional development of school-age children and adolescents.

[B59-behavsci-15-01362] Rashtchi M., Khoshnevisan B., Shirvani M. (2021). Integration of audiovisual input via TED-ED videos and language skills to enhance vocabulary learning. Mextesol Journal.

[B60-behavsci-15-01362] Richards J. C., Farrell T. S. C. (2005). Professional development for language teachers.

[B61-behavsci-15-01362] Roslim N., Azizul A. F., Nimehchisalem V., Abdullah M. H. T. (2021). Exploring movies for language teaching and learning at the tertiary level. Asian Journalof University Education.

[B62-behavsci-15-01362] Salape R. L., Maming J. B., Maravilla W. H. G. (2023). Content-based approach in developing listening comprehension in English language: Basis for proposed listening activities. International Journal of Multidisciplinary: Applied Business & Education Research.

[B63-behavsci-15-01362] Suarez M., Beato M. S. (2023). False memory in a second language: The importance of controlling the knowledge of word meaning. PLoS ONE.

[B64-behavsci-15-01362] Suhendi A., Purwarno P. (2018). Constructivist learning theory: The contribution to foreign language learning and teaching. AICLL: Annual International Conference on Language and Literature.

[B65-behavsci-15-01362] Suleiman Al Qunayeer H. (2021). An investigation of the relationship between reading comprehension, vocabulary knowledge, and English language proficiency level of Saudi EFL learners. Advances in Language and Literary Studies.

[B66-behavsci-15-01362] Swinburne Romine R., Schuster J., Karvonen M., Thompson W. J., Erickson K., Simmering V., Bechard S. (2025). Learning maps as cognitive models for instruction and assessment. Education Sciences.

[B68-behavsci-15-01362] *Universal Lithuanian Encyclopedia* (2025). Visuotinė lietuvių enciklopedija.

[B67-behavsci-15-01362] *Using Film and Media in the Language Classroom: Reflections on Research-Led Teaching* (2019). Using film and media in the language classroom: Reflections on research-led teaching.

[B69-behavsci-15-01362] Vilkienė L. (2011). Dvikalbis ugdymas Lietuvoje: Už ar prieš? [Bilingual education in Lithuania: For or against?]. Kalba Ir Kontekstai.

[B70-behavsci-15-01362] Vu T. B. H. (2023). Teaching English speaking skills: An investigation into Vietnamese EFL teachers’ beliefs and practices. Issues in Educational Research.

[B71-behavsci-15-01362] Vygotsky L. S. (1978). Mind in society: The development of higher psychological processes.

[B72-behavsci-15-01362] *Vygotsky’s Theory—An Overview Science Direct Topics* (2020). Vygotsky’s theory—An overview science direct topics.

[B73-behavsci-15-01362] Wang Z. (2023). The relationship between Chinese EFL learners’ foreign language writing enjoyment and writing vocabulary strategy. Theory & Practice in Language Studies (TPLS).

[B74-behavsci-15-01362] Weinstock D. M. (2022). The political ethics of bilingual education. Handbook of Philosophy of Education.

[B75-behavsci-15-01362] Woolfolk A. E. (1993). Educational psychology.

[B76-behavsci-15-01362] Yangin Ersanli C. (2023). The effect of using augmented reality with storytelling on young learners’ vocabulary learning and retention: Artırılmış Gerçeklik ile Hikâye Anlatımının Çocuk Öğrenenlerin Sözcük Öğrenimi ve Hatırda Kalıcılığına Etkisi. Novitas-Royal.

[B77-behavsci-15-01362] Yani L. H. F. (2022). Students’ perception of using film as an English learning media.

[B78-behavsci-15-01362] Zayas-Martínez F., Estrada-Chichón J. L., Segura-Caballero N. (2024). Pre-service CLIL teachers’ conceptions on bilingual education: Impact of initial training on the development of their teaching skills. Education Sciences.

[B79-behavsci-15-01362] Žydžiūnaitė V., Sabaliauskas S. (2017). Kokybiniai tyrimai: Principai ir metodai [Qualitative research: Principles and methods].

